# Thermally Reduced Graphene Oxide/Carbon Nanotube Composite Films for Thermal Packaging Applications

**DOI:** 10.3390/ma13020317

**Published:** 2020-01-10

**Authors:** Guang-jie Yuan, Jie-Fei Xie, Hao-Hao Li, Bo Shan, Xiao-Xin Zhang, Johan Liu, Long Li, Ying-Zhong Tian

**Affiliations:** 1 Shanghai Key Laboratory of Intelligent Manufacturing and Robotics, School of Automation and Mechanical Engineering, Shanghai University, Shanghai 200444, China; guangjie@shu.edu.cn (G.-j.Y.); lil@shu.edu.cn (L.L.); 2 Sino-Sweden Microsystem Integration Technology (SMIT) Center, School of Automation and Mechanical Engineering, Shanghai University, Shanghai 201800, China; okxjf@shu.edu.cn (J.-F.X.); lihaoer@i.shu.edu.cn (H.-H.L.); tonybo@i.shu.edu.cn (B.S.); zhangxx0914@shu.edu.cn (X.-X.Z.); johanliu@shu.edu.cn (J.L.); 3 Electronics Materials and Systems Laboratory, Department of Microtechnology and Nanoscience (MC2), Chalmers University of Technology, SE-412 96 Goteborg, Sweden

**Keywords:** graphene, carbon nanotubes, composite film, thermal interface materials

## Abstract

Thermally reduced graphene oxide/carbon nanotube (rGO/CNT) composite films were successfully prepared by a high-temperature annealing process. Their microstructure, thermal conductivity and mechanical properties were systematically studied at different annealing temperatures. As the annealing temperature increased, more oxygen-containing functional groups were removed from the composite film, and the percentage of graphene continuously increased. When the annealing temperature increased from 1100 to 1400 °C, the thermal conductivity of the composite film also continuously increased from 673.9 to 1052.1 W m^−1^ K^−1^. Additionally, the Young’s modulus was reduced by 63.6%, and the tensile strength was increased by 81.7%. In addition, the introduction of carbon nanotubes provided through-plane thermal conduction pathways for the composite films, which was beneficial for the improvement of their through-plane thermal conductivity. Furthermore, CNTs apparently improved the mechanical properties of rGO/CNT composite films. Compared with the rGO film, 1 wt% CNTs reduced the Young’s modulus by 93.3% and increased the tensile strength of the rGO/CNT composite film by 60.3%, which could greatly improve its flexibility. Therefore, the rGO/CNT composite films show great potential for application as thermal interface materials (TIMs) due to their high in-plane thermal conductivity and good mechanical properties.

## 1. Introduction

With the development of high-power electrical and electronic products, the heating problem of electronic devices has become increasingly serious; it restricts the lifetime, reliability and future development of electronic components [1,2]. Generally, the lifetime of transistors can be increased by one order of magnitude when the temperature of the hot spot is reduced by 20 °C [3,4,5,6]. In the real chip packaging structure, the actual contact is largely limited by the rough surfaces of the heat sink, heat spreader and chip [7,8]. Commonly, there is only 1–2% physical contact among the components, while the other space is filled with air [9,10,11]. To solve this issue, thermal interface materials (TIMs) are generally used to fill the space to improve the efficiency of heat dissipation. However, common TIMs only achieve a thermal conductivity of approximately 1~5 W m^−1^ K^−1^ at room temperature with a large filling ratio (approximately 70%) of the filler [11,12]. Some traditional TIMs still have thermal stability, thermal stress and other related issues, which make it increasingly difficult to meet the requirements of high-power device packaging. Therefore, it is extremely urgent to develop a novel TIM with high thermal conductivity.

Since the discovery of graphene in 2004, it has been the focus in various fields due to its unique two-dimensional (2D) structure [13]. The in-plane thermal conductivity of suspended monolayer graphene is reported to be from (4.84 ± 0.44) × 10^3^ to (5.30 ± 0.48) × 10^3^ W m^−1^ K^−1^ at room temperature, exceeding the bulk graphite limit of ca. 2000 W m^−1^ K^−1^ [14]. Therefore, graphene has been widely used as a novel carbon material to increase the thermal conductivity of polymer composites. However, due to the existence of van der Waals forces among the layers of the graphene film, the interlayer thermal resistance is generally high, so the through-plane thermal conductivity is much lower than the in-plane thermal conductivity, i.e., by more than two orders of magnitude [15,16]. Recently, graphene oxide (GO) has been widely used to synthesize reduced graphene oxide (rGO) films with a thermal annealing process, which could remove most of the oxygen-containing functionalities that disrupt the conjugated sp^2^ network of the basal plane of individual graphene sheets and lead to a significant drop in thermal conductivity [2]. However, the mechanical properties of the rGO films also dropped simultaneously because of random interlayer expansion during thermal decomposition of the functional sites [17]. This decrease caused the resulting materials to become brittle, which was not advantageous for further processing [18]. Recently, although some graphene-based films have been synthesized with high thermal conductivity and good mechanical properties, the cost is high for indispensable graphitization over 3000 °C [19]. Therefore, a novel low-cost graphene-based material must be strongly considered to avoid the graphitization process and still obtain high thermal conductivity and good mechanical properties.

Carbon nanotubes (CNTs) are novel carbon materials with excellent electrical, thermal and mechanical properties. Their thermal conductivity was measured to be more than 3000–3500 W m^−1^ K^−1^ at room temperature, and their tensile strength is 50–200 Gpa [20,21,22]. Some researchers have reported that the introduction of a small amount of CNTs can apparently improve the thermal conductivity and mechanical properties of composite materials [20,21]. Kim et al. found that adding 1 wt% single-walled CNTs to an epoxy resin increased the thermal conductivity by 25% [23]. In addition, Qian et al. found that CNTs with a mass fraction of 1% increased the elastic modulus of a polymer by 42% and the fracture stress by 25% [24]. He et al. also reported that, compared with pure epoxy, 0.5 wt% functionalized multiwalled CNTs could increase the tensile strength and impact strength of epoxy composites by 5.4% and 25.8%, respectively [25].

Because of the excellent performance of graphene and CNTs, rGO/CNT composite films have been widely constructed. Varshney et al. used nonequilibrium molecular dynamics simulations to investigate the thermal transport in one such novel architecture, a pillared graphene network nanostructure, which combined graphene and CNTs to create a three-dimensional network. The results showed that CNTs can highly improve the interfacial thermal conductance among adjacent graphene layers [26]. Pan et al. successfully prepared rGO/CNT composite films using a hydrothermal reduction method and found that their through-plane thermal conductivity increased from 0.055 to 0.091 W m^−1^ K^−1^ with increasing CNT content [27]. CNTs can be used to bridge adjacent graphene layers and promote phonon propagation in composite films [1].

While various attempts have been explored to construct rGO/CNT structural composite films for thermal management, few researchers have analyzed their mechanical properties. In this research, rGO/CNT composite films were prepared by thermal annealing of GO/CNT films without the graphitization process over 3000 °C, and their thermal conductivity and mechanical properties were analyzed. The main purposes of this study were (1) to systematically study the effect of the thermal annealing temperature on the microstructure, composition, thermal conductivity and mechanical properties of the rGO/CNT composite films and (2) to further explore the influence of the introduction of CNTs on the thermal conductivity and mechanical properties of the composite films.

## 2. Materials and Methods

**Figure 1:**
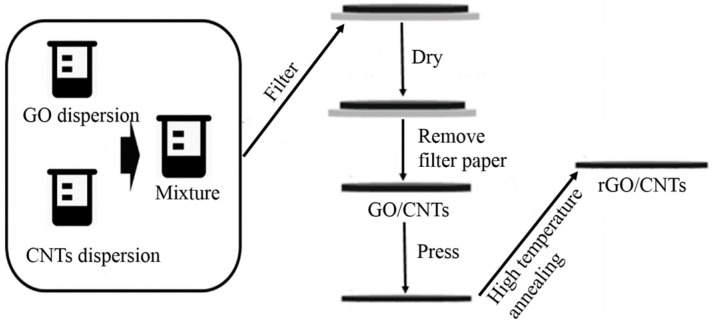
Illustration of the preparation process for the reduced graphene oxide/carbon nanotube (rGO/CNT) composite films.

The GO powder was purchased from Nanjing JCNANO Technology Co., Ltd (Nanjing, China), and the CNT dispersion was purchased from Nanjing XFNANO Materials Tech Co., Ltd (Nanjing, China). As shown in Figure 1, the GO powder was add to an appropriate amount of deionized water and stirred for 30 min to obtain the GO dispersion at a speed of 400 r min^−1^. The CNT dispersion with a mass of 1 wt% was added to the obtained GO dispersion, and the mixture was stirred for 30 min at a speed of 400 r min^−1^. Then, the GO/CNT composite films were prepared by vacuum-assisted flow filtration of the GO/CNT suspension through a nylon-66 membrane filter. The GO/CNT composite films were placed in a drying oven, baked at 50 °C for 1 h and then baked at 120 °C for 2 h. Afterwards, the dried composite films were peeled off and pressed on a tablet machine at a pressure of 190 MPa for 30 min to ensure the compactness of the internal structure of the GO/CNT composite films. Finally, to obtain the rGO/CNT composite films, the GO/CNT composite films were placed in a tube furnace and annealed at 1100, 1200, 1300 and 1400 °C for 2 h. The thickness of GO/CNT composite films was measured to be about 50 μm. The annealing process was as follows: First, the temperature of the tube furnace was increased from room temperature to 400 °C at a rate of 5 °C min^−1^ and then kept at 400 °C for 30 min to make the GO/CNT reduction process relatively gentle. Second, the temperature was increased from 400 to 1100, 1200, 1300 and 1400 °C at the rate of 5 °C min^−1^, and the reduction temperature was maintained for 2 h. To avoid the oxidation of rGO, the annealing process was carried out under argon, and its flow rate was set at 70 mL min^−1^. Finally, the temperature was reduced to room temperature at a cooling rate of 5 °C min^−1^.

Field emission scanning electron microscopy (FESEM; Merlin Compact) was used to characterize the cross sections of the composite films. To analyze the microstructure of the composite films, a Fourier transform infrared spectrometer (FT-IR; Nicolet iS50, Thermo Fisher Scientific, USA), a Raman spectrophotometer (INVIA, Renishaw, UK), and X-ray diffraction (XRD; D/MAX2500V+/PC, Rigaku, Japan) were used. X-ray photoelectron spectroscopy (XPS; ESCALAB250Xi, Thermo Fisher Scientific, UK) was used to analyze the chemical structures and compositions of the composite films. A laser flash thermal analyzer (Netzsch LFA 447, Germany) and differential scanning calorimetry (DSC; Mettler Toledo DSC1, Switzerland) were used to measure the thermal diffusivity (α) and specific heat capacity (C) of the composite films, respectively. The thermal conductivity of the composite films was generally calculated using Equation (1):

(1) λ=α×c×ρ

where λ and ρ are the thermal conductivity and density of the composite films, respectively. In addition, the Young’s modulus of the composite films was obtained by the nanoindentation method (Tribo Indenter, Hysitror, USA), and their tensile strength was measured by an electronic universal testing machine (AGS-X500N, Shimadzu, Japan).

## 3. Results and Discussion

### 3.1. Structural Characterization

**Figure 2:**
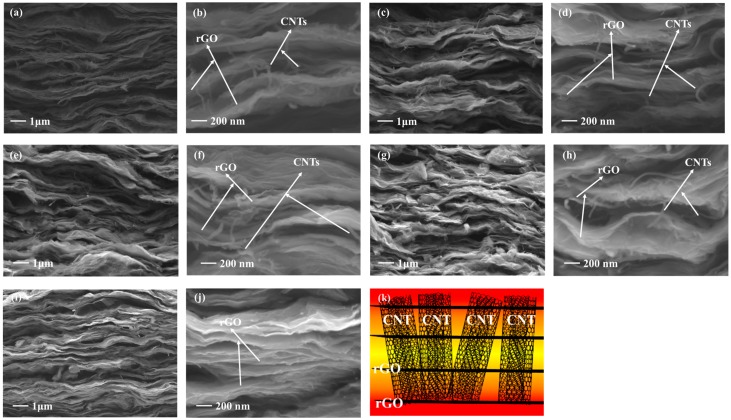
SEM cross-sectional images of the rGO/CNT composite films with different annealing temperatures: (**a**) Low magnification at 1400 °C, (**b**) high magnification at 1400 °C, (**c**) low magnification at 1300 °C, (**d**) high magnification at 1300 °C, (**e**) low magnification at 1200 °C, (**f**) high magnification at 1200 °C, (**g**) low magnification at 1100 °C and (**h**) high magnification at 1100 °C; SEM cross-sectional images of the rGO film after annealing at 1400 °C: (**i**) Low magnification and (**j**) high magnification; (**k**) schematic illustration of the rGO/CNT composite film.

Figure 2a–h shows the cross-sectional images of the rGO/CNT composite films with different annealing temperatures. The rGO layers exhibit a distinct layered structure and were mainly distributed in the horizontal direction. This special structure allows for the excellent in-plane thermal conductivity of the composite films [28,29]. Compared with the rGO film after annealing at 1400 °C as shown in Figure 2i,j, a certain amount of CNTs was present in the rGO/CNT composite films, and they connected the graphene layers with each other. They might provide longitudinal connections and heat conduction paths among the graphene layers, as shown in Figure 2k.

**Figure 3:**
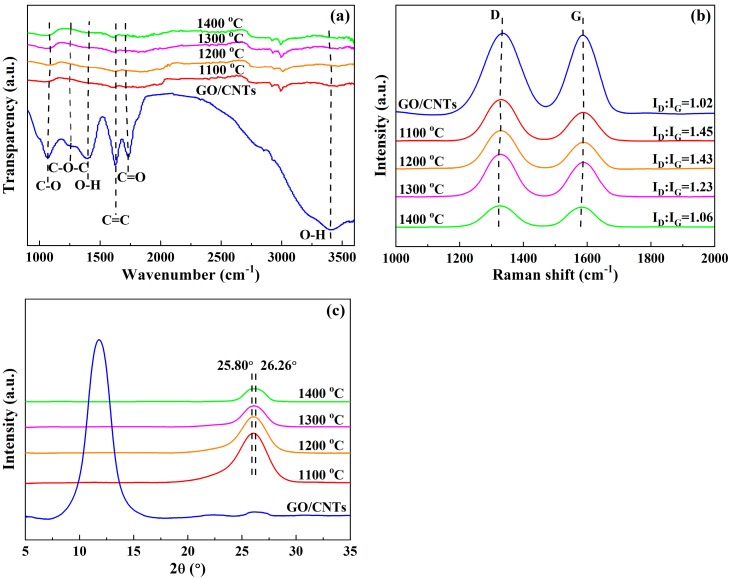
(**a**) FT-IR spectra; (**b**) Raman spectral evolution of D and G bands; and (**c**) XRD patterns of rGO/CNT composite films with different annealing temperatures.

As shown in the FT-IR spectra (Figure 3a), the stretching vibration peaks of C-O, C-O-C and C=O at approximately 1060, 1250 and 1731 cm^−1^, respectively, are almost invisible after annealing at 1100~1400 °C. In addition, the hydroxyl (O-H) band at approximately 3375 cm^−1^ almost disappeared after annealing. These changes indicated that most of the oxygen-containing functional groups were removed after the thermal annealing treatment [30,31]. For the GO film before annealing, the peak located near 1620 cm^−1^ might be related to oxygen surface compounds, ring vibrations throughout the carbon skeleton or the HOH bending vibrations also appearing in a very close rang of wavenumbers [13]. After annealing, the full width at half maxima (FWHM) was apparently reduced, and it might be caused by the removal of oxygen surface compounds or H_2_O. Besides, the stretching vibration peak of δC=C was still present at approximately 1620 cm^−1^, indicating that the conjugated C=C was effective repaired in sp^2^ graphitic region [29]. As shown in the Raman spectra (Figure 3b), the value of I_D_:I_G_ of the composite films significantly increased from 1.02 for the original GO/CNT composite film to 1.45 after annealing at 1100 °C. This change could be explained by the increment in the structural defects of the rGO/CNT composite films because a large amount of oxygen in epoxy groups was converted into CO and/or CO_2_ species [32,33]. Additionally, when the annealing temperature increased from 1100 to 1400 °C, the value of I_D_:I_G_ gradually decreased from 1.45 to 1.06, which was attributable to the promotion of new sp^2^ clusters and the recovery of more sp^2^ C=C bonds in the graphite lattice [34]. This result indicated that a higher annealing temperature could effectively reduce the internal structural defects of the rGO/CNT composite films. As shown in the XRD patterns (Figure 3c), the characteristic peaks of GO corresponding to approximately 12° almost disappeared, while the characteristic peaks of graphene appeared at approximately 26°, which confirmed that the GO was mostly reduced to graphene. As the annealing temperature increased from 1100 to 1400 °C, the position of the graphene (002) peaks slightly shifted to the right, which indicated that the percentage of graphene gradually increased in the composite films. As shown in Table 1, the interlayer distance of the rGO/CNT composite films gradually decreased from 0.345 to 0.339 nm as the annealing temperature increased. Generally, the interlayer distance of GO is larger than that of graphene [35]. This change indicated that when the annealing temperature increased, more oxygen-containing functional groups decomposed, which resulted in a higher percentage of graphene and tighter interlayers in the rGO/CNT composite films.

**Figure 4:**
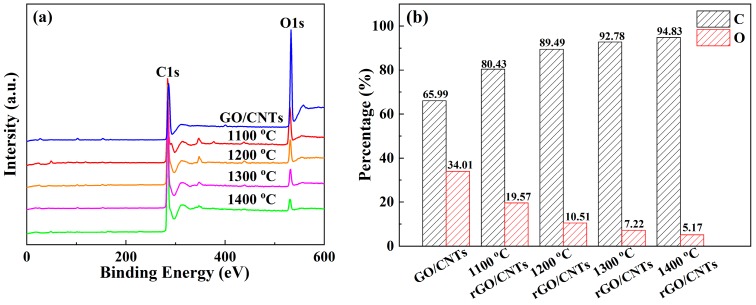
(**a**) XPS spectra of the rGO/CNT composite films; (**b**) the percentage of carbon and oxygen in the composite films with different annealing temperatures.

Figure 4a shows the XPS spectra of the rGO/CNT composite films after annealing at different temperatures. Figure 4b shows the quantitative analysis of the percentages of C and O in the composite films at different annealing temperatures. For the original GO/CNT composite film, the percentage of O was 34.01%, which indicated that in addition to a certain amount of C=C/C-C bonds, the composite films also had mostly oxygen-containing functional groups, such as carbonyl and hydroxyl groups [36]. As shown in Figure 4a,b, when the annealing temperature increased from 1100 to 1400 °C, the percentage of C increased from 80.43% to 94.83%, and the percentage of O apparently reduced from 19.57% to 5.17%. This change means that the content of C=C/C-C bonds increased and more oxygen-containing functional groups thermally decomposed in the rGO/CNT composite films as the annealing temperature increased, which confirmed the FT-IR, Raman and XRD results in Figure 3a–c.

### 3.2. Thermal Property Characterization

**Figure 5:**
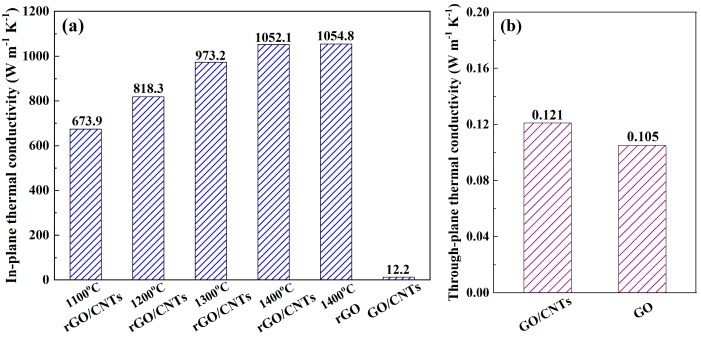
(**a**) In-plane thermal conductivity of the rGO/CNT composite films with different annealing temperatures compared with that of the rGO film after annealing at 1400 °C; (**b**) through-plane thermal conductivity of the GO/CNT composite film compared with that of the GO film.

Figure 5a shows the in-plane thermal conductivity of the rGO/CNT composite films after annealing at different temperatures compared with that of the rGO film after annealing at 1400 °C. Compared with the original GO/CNT composite film, the in-plane thermal conductivity of the rGO/CNT composite film dramatically increased from 12.2 to 673.9 W m^−1^ K^−1^ after annealing at 1100 °C. In addition, when the annealing temperature increased, the in-plane thermal conductivity also gradually increased. This increase was attributed to reconnection of the original sp^2^ clusters by newly formed smaller sp^2^ domains, and the phonon transport was dominated by percolation [17,27]. In addition, the in-plane thermal conductivity of the rGO/CNT composite film was measured to be 1052.1 W m^−1^ K^−1^ after annealing at 1400 °C, which far exceeded that of copper (≈400 W m^−1^ K^−1^) and was higher than that of the high-density aligned carbon nanotube paper (≈776 W m^−1^ K^−1^) [37,38]. It was also quite close to the in-plane thermal conductivity of the rGO film (1054.8 W m^−1^ K^−1^), which indicated that the addition of 1% CNTs had little effect on the in-plane thermal conductivity of the rGO/CNT composite films that maintained the initial high in-plane orientations.

To confirm that CNTs provided the exact longitudinal heat conduction paths for the transport of phonons in the rGO/CNT composite films, their through-plane thermal conductivity was commonly measured. However, the thickness of the synthesized rGO/CNT composite films was in the range of several tens of micrometers, which could result in great error in the measurement of the through-plane thermal conductivity. Generally, the distribution of CNTs in the original thick GO/CNT composite film was almost the same as that in the synthesized rGO/CNT composite film. In addition, compared with the rGO/CNT composite films, the role of CNTs was much easier to detect in the original GO/CNT composite film due to the great contrast in the thermal conductivity of GO and the CNTs. Therefore, the through-plane thermal conductivity of the 450-μm-thick GO/CNT composite film was measured to confirm the role of the CNTs as heat conduction paths for the longitudinal transport of phonons. As shown in Figure 5b, compared to the GO film, the through-plane thermal conductivity of the GO/CNT composite film increased by 20%, which suggested that the CNTs acted as bridges among the GO layers and contributed to the heat conduction paths along the through-plane direction [1]. As mentioned above, because the CNTs had a similar distribution in the original GO/CNT and synthesized rGO/CNT composite films, the CNTs could also provide exact longitudinal heat conduction paths in the rGO/CNT composite films, which is beneficial for the improvement of their through-plane thermal conductivity.

### 3.3. Mechanical Property Characterization

**Figure 6:**
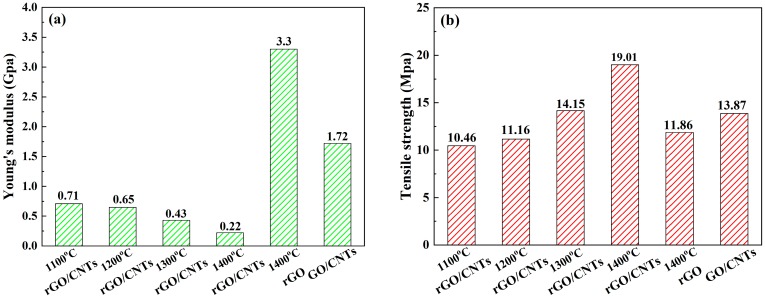
Mechanical properties of the rGO/CNT composite films with different annealing temperatures compared with those of the rGO film after annealing at 1400 °C: (**a**) Young’s modulus; (**b**) Tensile strength.

Figure 6a shows the Young’s modulus of the rGO/CNT composite films with different annealing temperatures compared with that of the rGO film after annealing at 1400 °C. Compared with the original GO/CNT composite film, the Young’s modulus of the rGO/CNT composite films decreased from 1.72 to 0.71 Gpa after annealing at 1100 °C. In addition, as the annealing temperature increased from 1100 to 1400 °C, the Young’s modulus also gradually decreased from 0.71 to 0.22 Gpa. This change could be explained by the fact that as the annealing temperature increased, the carbon bonds that formed between the interconnected graphene layers and the defects also decreased, which resulted in a reduction in the Young’s modulus [29,35,39]. Additionally, after annealing at 1400 °C, the Young’s modulus of the rGO film was 15 times that of the rGO/CNT composite film, which indicated that the addition of CNTs could greatly improve the flexibility of the rGO/CNT composite film [40].

Figure 6b shows the tensile strength of the rGO/CNT composite films with different annealing temperatures compared with that of the rGO film after annealing at 1400 °C. When the annealing temperature increased from 1100 to 1400 °C, the tensile strength of the composite films increased from 10.46 to 19.01 MPa, which indicated that the tensile strength improved as the annealing temperature increased. This increase was attributed to the gradual restoration of orderly stacks of graphene layers upon removal of the oxygen-containing functional groups, decreased interlayer spacing and enhanced interlayer contact of the rGO layers [41]. As shown in Figure 6b, the tensile strength of the rGO/CNT composite film was 19.01 MPa after annealing at 1400 °C, which was 60.3% higher than that of the rGO film (11.86 MPa). This difference indicated that CNTs play an important role in the enhancement of the composite film tensile strength. The graphene layers might connect with the bulging parts of the CNTs at the overlap joint, forming a special interlocking-tile structure, and the remaining portion of the graphene layers stack on one another to maintain a flexible structure [17,42]. Compared with the rGO film, the rGO/CNT composite films have a larger interaction surface, which also resulted in the improvement in their tensile strength [17,42].

## 4. Conclusions

The rGO/CNT composite films had a distinct layered structure in the horizontal direction, which provided their excellent in-plane thermal conductivity. When the annealing temperature increased, their in-place thermal conductivity and mechanical properties significantly improved. In addition, a certain amount of CNTs among the graphene layers in the composite films could provide through-plane thermal conduction pathways. Moreover, CNTs also played an important role in enhancing the mechanical properties of the rGO/CNT composite films. While only 1% of CNTs were added to the composite films, the CNTs could apparently reduce the Young’s modulus and improve the tensile strength and flexibility. In summary, compared with the rGO film, the rGO/CNT composite film had comparable in-plane thermal conductivity and better mechanical properties, indicating it has potential as a TIM for thermal packaging applications.

## Figures and Tables

**Table 1:** Interlayer spacing of the rGO/CNT composite films with different annealing temperatures.

Samples	GO/CNTs	1100 °C	1200 °C	1300 °C	1400 °C
Grazing angle θ (°)	5.90	12.90	13.05	13.09	13.13
Interlayer spacing d (nm)	0.749	0.345	0.341	0.340	0.339
